# Properties of Gd-Doped Sol-Gel Silica Glass Radioluminescence under Electron Beams

**DOI:** 10.3390/s22239248

**Published:** 2022-11-28

**Authors:** Daniel Söderström, Oskari Timonen, Heikki Kettunen, Risto Kronholm, Hicham El Hamzaoui, Bruno Capoen, Youcef Ouerdane, Adriana Morana, Arto Javanainen, Géraud Bouwmans, Mohamed Bouazaoui, Sylvain Girard

**Affiliations:** 1 Accelerator Laboratory, Department of Physics, University of Jyvaskyla, FI-40014 Jyvaskyla, Finland; 2 Univ-Lille, CNRS, UMR 8523-PhLAM-Physique des Lasers Atomes et Molécules, 59000 Lille, France; 3 Univ Lyon, Laboratoire H. Curien, UJM-CNRS-IOGS, 18 Rue du Pr. Benoît Lauras, 42000 Saint-Etienne, France

**Keywords:** dosimetry, electron accelerator, optical fiber, point dosimeter, pulsed electron beam, radiation-induced attenuation, radiation-induced luminescence

## Abstract

The radiation-induced emission (RIE) of Gd^3+^-doped sol–gel silica glass has been shown to have suitable properties for use in the dosimetry of beams of ionizing radiation in applications such as radiotherapy. Linear electron accelerators are commonly used as clinical radiotherapy beams, and in this paper, the RIE properties were investigated under electron irradiation. A monochromator setup was used to investigate the light properties in selected narrow wavelength regions, and a spectrometer setup was used to measure the optical emission spectra in various test configurations. The RIE output as a function of depth in acrylic was measured and compared with a reference dosimeter system for various electron energies, since the dose–depth measuring abilities of dosimeters in radiotherapy is of key interest. The intensity of the main radiation-induced luminescence (RIL) of the Gd^3+^-ions at 314 nm was found to well represent the dose as a function of depth, and was possible to separate from the Cherenkov light that was also induced in the measurement setup. After an initial suppression of the luminescence following the electron bunch, which is ascribed to a transient radiation-induced attenuation from self-trapped excitons (STEX), the 314 nm component was found to have a decay time of approximately 1.3 ms. An additional luminescence was also observed in the region 400 nm to 600 nm originating from the decay of the STEX centers, likely exhibiting an increasing luminescence with a dose history in the tested sample.

## 1. Introduction

Silica glass doped with Gd^3+^-ions fabricated through the sol–gel process has been shown to be suitable for use in dosimeters for X-ray beams [1], proton beams [2], and electron beams [3]. The radiation-induced luminescence (RIL) generated in the doped silica glass has in these studies been shown to be proportional to the ionizing dose on the tested samples, by studying the light generated in the doped glass, which was transported away from the irradiation area through an optical fiber, fusion-spliced to the doped glass.

The RIL response under steady-state irradiation was studied using X-rays in [1]. There, the response was found to be at least linear in the dose rate range 125 μGy(SiO_2_)/s to 12.25 Gy(SiO_2_)/s. In the study using proton irradiation [2], the sample was irradiated by the proton beams of energies between 6 MeV and 63 MeV, with dose rates from 0.02 Gy/s to 0.30 Gy/s. The dose profile of the proton beams in water was also investigated in the study, along with the samples doped with Ce^3+^-ions and Cu^+^-ions. The doped glass samples’ ability to resolve the proton Bragg peak was compared with a reference Markus chamber, where the Gd^3+^-doped sample came close to the performance of the Markus chamber, and showed better capabilities in this aspect than the samples with other dopants. The response to the electron radiation of doped silica glasses was tested in [3], where samples doped with Ce^3+^-ions, Cu^+^-ions, and Gd^3+^-ions were investigated. In that study, a pulsed beam was used and the RIL response to the variations of the electron bunch sizes were studied. The tested samples showed a linear RIL response in the dose-per-bunch range 10^−5^ Gy/bunch–1.5 × 10^−2^ Gy/bunch, for the 3 μs long electron bunches. The RIL of Gd^3+^-ions is ascribed to the transition between the ^6^P_7/2_ and ^8^S_7/2_ levels [1,2,4]. This transition produces a narrow emission peak at 314 nm [1,3,4]. The decay time of this transition is fairly long, and has been measured to be 1.80 ms in [5] for the comparable doping level of 0.05 mol%, and to 1.35 ms in [1], a similar sample as the one tested in this paper, with a dopant level of 0.1 wt%≈0.04 mol%.

SiO_2_ also has radiation-induced absorption (RIA) and RIL bands, which are not related to the Gd^3+^-dopant ions. Studies of the transient RIA was performed on amorphous SiO_2_ samples using pulsed electron beams in [6], where transient absorption bands were observed at 5.3 eV (234 nm), and at 4.2 eV (295 nm). These absorption bands are ascribed to the metastable centers formed after electron–hole pair generation, called self-trapped excitons (STEXs) [6,7]. The STEX centers in [8] were found to be formed by an Si-O bond breaking when an electron was excited, and the lattice subsequently distorting, trapping the excited electron and the hole, respectively, on the broken apart Si and O atoms. The STEXs causing the transient RIA were in [6] found to have a luminescent decay by emission energies around 2.4 eV (517 nm), also exhibiting a blue-shift over the time of the decay. The luminescence in [9] was measured to have a peak energy around 2.8 eV, and a decay time close to 1 ms when the sample was cooled down to temperatures below 170 K. At temperatures above 170 K, the decay time was found to decrease with increasing temperature.

An important quality for dosimeter systems used in radiotherapy contexts is the ability to represent dose as a function of depth in water, since the dose deposited in human tissue is the key parameter for radiotherapy. For the Gd^3+^-doped silica glass dosimeters studied in this paper, the ability to measure the dose–depth curves of protons in water has been previously demonstrated [2]. The corresponding dose–depth curves of electron beams measured with this type of sample have not yet been studied in the literature to the best of the authors’ knowledge.

When measuring the light output of an optical fiber irradiated by electrons at a depth in water or another material, a signal from Cherenkov light will be induced with increasing intensity as a function of depth, as the electrons scatter more frequently at larger angles [10]. The depth profile of emitted Cherenkov light in an optical fiber differs from the depth profile of an ionizing dose. The dose–depth profile from electron beams displays an initial increase due to the generation of secondary particles up to the maximum dose value, then a decrease of deposited dose due to the decrease in the primary beam intensity, and finally, a longer tail by induced X-ray secondaries (see, e.g., [11] for further descriptions).

The dose–depth profiles of electron beams in an acrylic phantom, measured using a Gd^3+^-doped silica glass sample, were studied in this paper. Acrylic is commonly used as a substitute to water in the dosimetric measurements of clinical beams due to its similar properties to water [12], and thus, provides a good understanding of the behavior of the Gd^3+^-doped sample under electron beams in tissue for medical applications. The sample was also studied under electron beams at varying angles relative to the sample to for further study the properties of the induced Cherenkov emission. The properties of the RIL from the Gd^3+^-doped glass was also investigated in narrow wavelength regions selected with a monochromator setup, where the decay times of the RIL emissions were measured.

## 2. Materials and Methods

### 2.1. Tested Sample

A Gd^3+^-doped sol–gel silica glass rod was tested. The rod was 1 cm-long and had a diameter of approximately 500 μm, and a dopant concentration of 0.1 wt% Gd^3+^-ions. The descriptions of the fabrication process of this type of sample were found in, e.g., [13,14]. The Gd^3+^-doped rod, drawn at a temperature of approximately 2000 °C, was fusion-spliced to a 500 μm pure-silica core multimode optical fiber, with a numerical aperture of 0.4. This optical fiber was used to transport the RIL to the signal analysis and read-out systems described in Section 2.2.

### 2.2. Test Setup

A monochromator was used to select specific wavelength regions of the RIL for analysis. The monochromator was a 996 mm focal length Fastie–Ebert type monochromator with a 2200 grooves/mm holographic diffraction grating, which was further described in [15]. The RIL-light from the 500 μm transport fiber was sent to the monochromator through two aspheric lenses, which converted the numerical aperture and focused the light to fit a fiber bundle which led the light into the monochromator.

At the output end of the monochromator, the light was detected by a Hamamatsu R9880U-110 photomultiplier tube (PMT) [16]. The spectral resolution of a monochromator is partly determined by the width of its output aperture, and the total photon flux measured at the output is determined by the aperture area. Typically, a narrow slit is used in front of the output aperture in order to achieve the desired spectral resolution, at the expense of measured light intensity. However, in order to obtain a detectable light signal in these experiments, no exit slit was used and the detection resolution was limited by the 8 mm-diameter photosensitive area of the PMT window, resulting in an instrumental FWHM of approximately 3.5 nm.

The output wavelength of the monochromator was selected by rotating the diffraction grating with a stepping motor. The stepper motor was driven by an ST-7128 Microstep Driver, and controlled by an Arduino UNO. The amount of steps taken was set with the measurement PC via a USB serial connection using Python. The same Python program also controlled the data transfer to the PC of the digitized PMT signal through a CAEN N6751 digitizer module [17].

The PMT signal was sent to a fast linear amplifier then to the digitizer. The electron beam used for the tests was pulsed (further described in Section 2.3), and the digitizer acquisition was controlled by a trigger signal coming from an external Si-diode detector in the electron beam. The induced signal from the Si diode when it was struck by an electron bunch was sent to a single channel analyzer, which generated a trigger signal transmitted to the digitizer. Upon the trigger signal arrival, the digitizer acquired a signal trace consisting of a predefined length of the PMT signal. The trace was up to 1 ms-long, with a sampling interval of 0.5 ns within the trace. This allowed the detection of emitted photons by the sample at a specific wavelength, and with a known timing relative to the electron bunch.

To take wider emission spectra, an Ocean Optics USB2000+ UV–VIS–ES spectrometer [18] was used. When the spectrometer was used, the end of the transport fiber was directly fixed by the spectrometer input window.

### 2.3. Irradiation Facility

The irradiation tests were carried out at the Radiation Effects Facility (RADEF), in the accelerator laboratory of the University of Jyväskylä, Finland. A Varian Clinac 2100C/D linear electron accelerator [3,19] was used to generate the electron beam for the experiments. Electrons with energies of 6, 9, 12, 16, and 20 MeV are available from the Clinac, along with 6 and 15 MV photon beams, consisting of photons with energy spectra, respectively, reaching up to 6 and 15 MeV. The photon beams are bremsstrahlung spectra generated by electrons with energies of 6 and 15 MeV impinging on a metal target.

This study focuses on electron beams, where the energies 6, 12, and 20 MeV were used. The dose rates of the electron beams can be set between 100 rad(H_2_O)/min and 1 krad(H_2_O)/min, corresponding to average dose rates of 17 mGy(H_2_O)/s–0.17 Gy(H_2_O)/s. The dose rate was monitored by an internal ionization chamber in the machine, which was calibrated against an external ionization chamber at the maximum dose–depth in water. The dose and dose rate levels set by the machine therefore refer to the dose in water.

When changing the dose rate, the electron bunch frequency is modulated, but the size of the bunches stays the same. A ten-fold increase in dose rate thus corresponds to a ten-fold higher bunch frequency. At the lowest dose rate setting from the machine, 100 rad(H_2_O)/min, the average electron bunch frequency is approximately 20 Hz. This means that the dose per electron bunch is approximately 0.83 mGy, and the instantaneous dose rate from the machine is close to 280 Gy/s during the approximately 3 μs-long electron bunches.

The Clinac at RADEF is an accelerator that was previously used in a hospital for radiotherapy purposes. The gantry is rotatable around the machine isocenter, which is the position at which the dosimetry calibration of the machine is performed, and the location where the tested sample was positioned.

### 2.4. Test Methodology

When using the monochromator setup, in combination with the PMT and digitizer, the lowest available dose rate of 100 rad(H_2_O)/min (17 mGy(H_2_O)/s) from the machine was always used. This was due to the long data traces saved through the digitizer (up to 1 ms), which was associated with a long data processing and transfer time. The digitizer was always busy when recording the data; thus, a higher bunch frequency would not have resulted in any benefits regarding the data collection rate.

The total light transfer efficiency from the doped sample to the PMT was not measured explicitly. The amount of emitted light on the PMT was, however, not large, and irradiation runs of approximately 1 h were utilized for each measurement point and test configuration. To be able to compare the results between different test configurations, the number of detected photon signals per saved digitizer trace were used as a comparable metric. Here, one trace in the digitizer corresponds to one electron bunch from the accelerator.

With the spectrometer setup, the dose rate from the electron accelerator that was used was instead the highest available, 1 krad(H_2_O)/min (0.17 Gy(H_2_O)/s). In this case, the acquired spectra were generated by summing up the emitted photons from the sample during 20 s-long time intervals. Three such intervals were used for each irradiation configuration, reaching 1 min of irradiation per tested configuration.

The deposited dose as a function of depth in acrylic was tested, and discussed in Section 3.2. This was performed by placing the sample in the machine isocenter on top of a 5 cm thick block of acrylic, and placing the sample and transport fiber between two sheets of acrylic with a thickness of 2 mm. The depth in acrylic was then varied by placing layers of acrylic sheets of 5 and 10 mm thickness on top of the sample.

**Figure 1:**
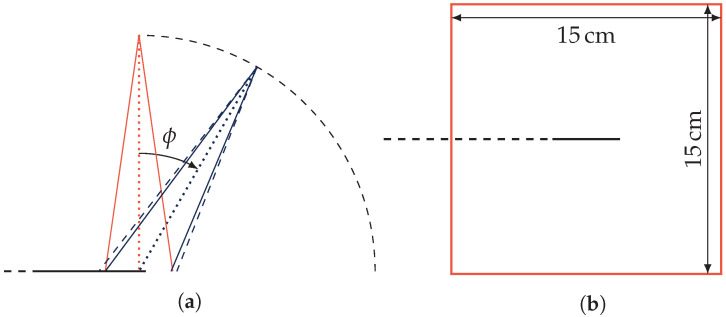
Schematic view of the beam tilting procedure. (**a**) View from the side, where ϕ is the tilting angle of the beam, and the sample with the transport fiber is marked by the horizontal black line ending in a dashed line. (**b**) View from the top, with the sample laying in the center of the beam window marked in orange, and the transport fiber directed to the left of the figure along the black dashed line. The beam tilting direction is towards the right.

Tests at different beam rotation angles were performed as well, as shown in Section 3.3. These were performed by suspending the sample freely in air, only supported by a thin plastic sheet. The amount of material surrounding the tested sample was minimized to lessen the scattering of electrons onto the sample from varying angles, potentially producing additional Cherenkov radiation. The transport fiber was oriented away from the beam tilting direction, which is schematically shown in Figure 1a, where ϕ is the tilt angle of the gantry.

A beam window size of 15 cm × 15 cm was used for the tests at the normal incidence angle (ϕ=0, and for dose–depth tests in acrylic), which is shown in Figure 1b, where the sample is seen in the center of the beam window. The total length of sample and transport fiber in the beam is thus 8 cm. After beam rotation, the beam area was changed so that the portion of the sample and transport fiber that was irradiated was always the same. This is shown in Figure 1a, where the 15 cm × 15 cm beam after rotation is drawn with a dashed line, and the corrected beam after rotation is marked by full blue lines.

## 3. Results and Discussion

### 3.1. RIL at Selected Wavelengths

**Figure 2:**
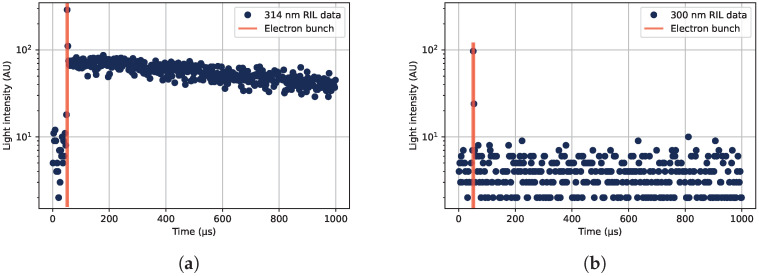
Examples of the time structure of the radiation-induced emission (RIE), as detected at different wavelengths under irradiation by a pulsed 20 MeV electron beam. (**a**) The radiation-induced luminescence (RIL) of the Gd^3+^-ions at 314 nm, along with the prompt radiation response during the electron bunch, marked with orange lines around 50 μs into the collected digitized data traces. (**b**) Detected photons at 300 nm, consisting of a prompt response but no visible RIL component.

From the digitized traces of collected photons in the PMT, histograms of the times of detected photons relative to the electron bunch can be constructed. From such histograms, the RIL can be seen, as shown in Figure 2. In Figure 2a, the luminescence of the main 314 nm emission line of Gd^3+^ is shown. If a different wavelength is selected from the monochromator, the RIL after the electron pulse is gone, but the prompt luminescence response during the electron bunch is still present, as shown for 300 nm in Figure 2b.

**Figure 3:**
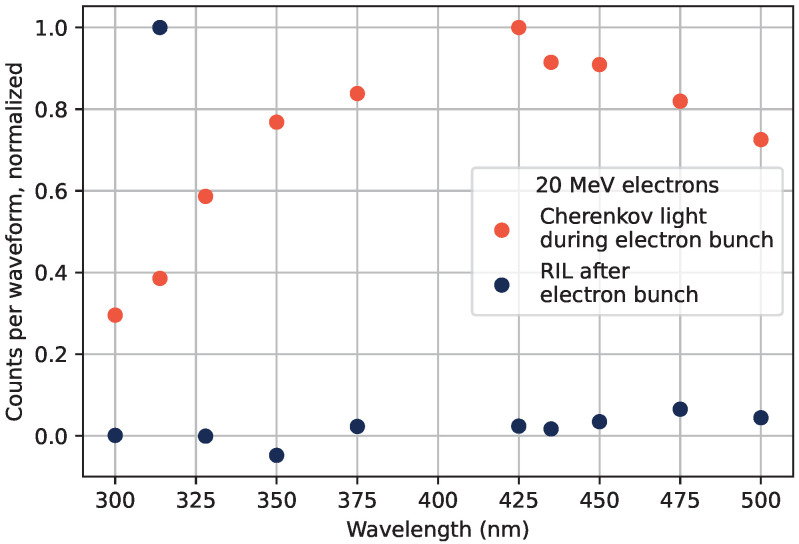
RIE spectra from 20 MeV electrons at a normal incidence angle without any water equivalent material above the sample. The luminescence detected after the electron bunches is shown in blue, and the prompt radiation response during the electron bunch in orange.

To generate the data shown in Figure 2, the sample was placed under the beam covered with a thin black plastic tube to shield the sample from outside light sources, and without any acrylic covering the sample. Under these irradiation conditions, the RIL was investigated at different wavelengths, which is shown in Figure 3. The prompt responses to the electron beam as well as the luminescence occurring after the electron bunch at different wavelengths are displayed in the figure. The different parts of the detected luminescence can be seen in Figure 2, where the time position of the electron bunch is marked in orange vertical lines, the RIL is present after the electron bunch, and the prompt response from Cherenkov radiation is obtained during the electron bunch.

The prompt response spectral shape marked with orange dots in Figure 3 is similar to what was observed in, e.g., [20] for Cherenkov radiation. In the figure, the PMT spectral sensitivity was accounted and corrected for. The Cherenkov emission spectrum commonly described by the Frank–Tamm formula [21,22] predicts a continuously growing emission at shorter wavelengths. This is not observed, as the material absorbance at shorter wavelengths in the UV spectrum is high [23]. The suppression of the short wavelengths of Cherenkov spectra is further discussed in, e.g., [24,25].

The RIL spectrum shown in blue in Figure 3 presents the expected dominating emission of the Gd^3+^-ions at the datum point at 314 nm. Further tendencies of emissions can be seen in the 450 nm–500 nm spectral region. This emission will be investigated and further discussed in Section 3.5.

### 3.2. Dose as a Function of Depth in Acrylic

**Figure 4:**
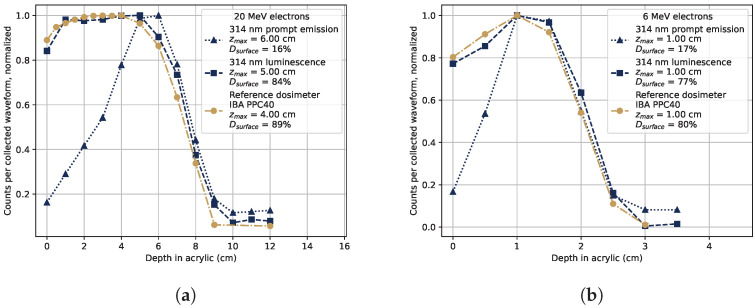
Generated light intensity in the sample as a function of depth in acrylic at 314 nm. The RIL after the electron pulse (squares) and the prompt light signal (triangles) were separated as in Figure 3. The response of a reference dosimeter, an IBA PPC40, is included in the figures as a gold dash-dotted line. The figure legends display the depth at which the maximal dose was recorded, $z_{max}$, and the surface dose (0 cm depth) fraction relative to the maximum dose as $D_{surface}$. (**a**) 20 MeV electron beam. (**b**) 6 MeV electron beam.

The deposited dose as a function of depth in acrylic is shown in Figure 4. The response to 20 MeV electrons is shown in Figure 4a, and for 6 MeV electrons in Figure 4b. The gold-colored lines in the figures represent measurements taken using a reference parallel plate ionization chamber dosimeter IBA PPC40 [26], and the blue lines are from measurements with the tested sample, where the monochromator was set to 314 nm to select the Gd^3+^ luminescence signal. The figure legends contain information of the depth of maximum dose (maximum signal level) $z_{max}$ measured by each method, as well as the fraction of dose (signal strength) at 0 cm of acrylic compared to that at $z_{max}$, denominated as the surface dose $D_{surface}$.

The same procedure as described for Figure 3 was used to separate the luminescence response after the electron bunch from the prompt signal caused by Cherenkov radiation. The RIL at 314 nm from the Gd^3+^-ions is able to represent the dose as a function of depth in water in a manner very similar to the reference dosimeter. The Cherenkov radiation induced in the sample has a different shape, caused by the increased scattering of the electrons at larger angles as the depth increases, which is also shown and discussed in [10]. Some parts of the prompt signal might originate from other sources than Cherenkov radiation, but the shape of the depth profile of the prompt emissions in Figure 4 and the wide emission spectrum in Figure 3 suggest that a large majority of the light originates from Cherenkov radiation.

**Figure 5:**
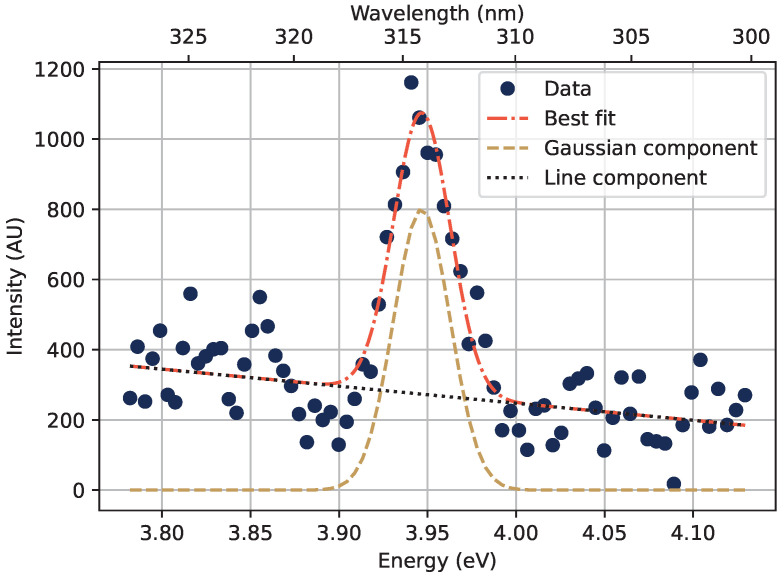
The wavelength region with the 314 nm emission peak from the Gd^3+^-ions as measured under 4 cm acrylic and 12 MeV electrons. The emission peak was approximated as a Gaussian over the Cherenkov radiation background, which in turn was approximated with a straight line in the region shown in the figure.

The light output from the sample as a function of depth in acrylic was also analyzed using a spectrometer, by obtaining emission spectra with various thicknesses of acrylic covering the sample. The procedure used for separating the RIL at 314 nm (3.95 eV) from the Cherenkov background is shown in Figure 5, where the measured RIL peak is approximated as a Gaussian, the Cherenkov spectrum in the narrow region around the RIL peak is approximated as a straight line, and the total signal is fitted to the sum of those two components.

**Figure 6:**
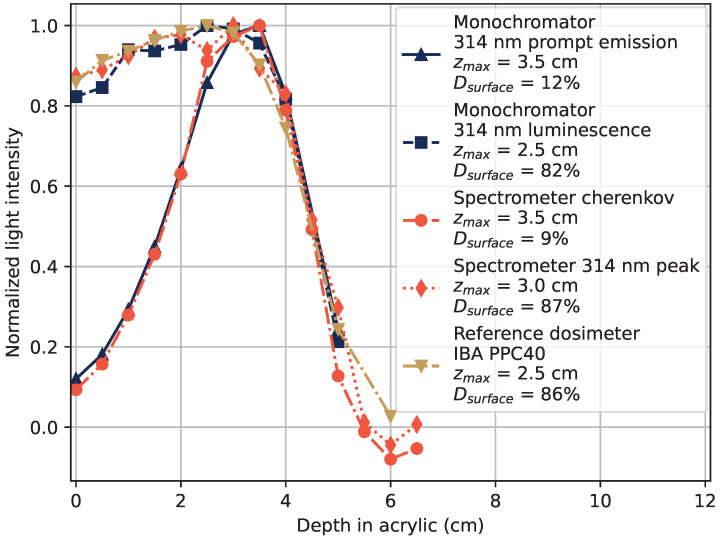
Dose–depth curves in acrylic of 12 MeV electrons obtained with the digitized PMT signal and monochromator set to 314 nm as shown in blue, using optical spectrometer in orange, and IBA PPC40 dosimeter in gold.

The dose–depth curve of 12 MeV electrons is shown in Figure 6. Results from measurements with an optical spectrometer is included, where the size of the RIL peak at 314 nm is extracted as the signal above the Cherenkov background in measured emission spectra at each tested depth of acrylic, by the procedure exemplified in Figure 5. The Cherenkov radiation component is approximated as the total amount of detected light which is not in the 314 nm peak, in the full measurement interval of the spectrometer from 180 nm to 875 nm.

Both methods of light detection and separation between Cherenkov light and RIL from Gd^3+^-ions, using a spectrometer and monochromator, show that the Gd^3+^ luminescence well represents the deposited dose in the material. They also show that Cherenkov radiation is present and needs to be taken into account, since the radiation profiles by Cherenkov radiation do not follow the profile of deposited dose.

### 3.3. Beam Tilting Tests

For further investigation of the Cherenkov component of the emitted light, irradiation with a tilted beam was performed. The beam tilting was performed by rotating the accelerator gantry around the sample, in the direction away from the transport fiber as described further in Section 2.4. By this procedure, the Cherenkov radiation through the transport fiber should increase with the beam angle ϕ, up to a maximum emission located at the Cherenkov emission angle $\theta =90^{\circ }-\phi_{max}$, when the induced emission is directed along the transport fiber. The Cherenkov emission angle can be calculated as

(1) $$\cos\theta =\frac{1}{\beta \;n(\lambda )},$$

where β is the relative velocity of the charged particle inducing the Cherenkov radiation, and n(λ) is the refractive index of the material, which varies with the wavelength λ of the emitted light. This relation is discussed further in, e.g., [22].

All electrons do not arrive in a parallel orientation at the sample, since the beam is narrow as it leaves the accelerator and spreads with an opening angle towards the irradiated sample. The electrons will thus have a distribution of angles in relation to the sample and transport fiber orientation. This angular distribution of electrons will cause a broadening of the peak around the Cherenkov emission angle in the detected light intensity as a function of the beam tilting angle. Further broadening of the peak around the Cherenkov angle will be caused by the acceptance angle of the fiber, since photons in a range of scattering angles will be transmitted through the fiber.

**Figure 7:**
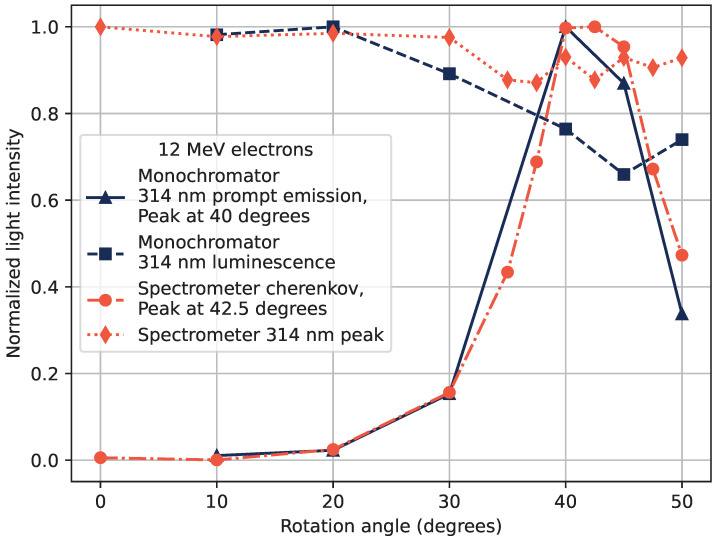
Detected light intensity as a function of the beam tilting angle of a 12 MeV electron beam obtained with digitized PMT signal and monochromator set to 314 nm shown in blue, and with optical spectrometer shown in orange.

The effect of the electron beam tilt angle on the light output is shown in Figure 7 using a 12 MeV electron beam. Similarly to Figure 6, Figure 7 shows the results from the measurements with both the optical spectrometer and the monochromator set to 314 nm. The effects of rotation on the different path-lengths of electrons in the sample and transport fiber, and variations in the beam window size were taken into account and corrected for in the figure.

The measured Cherenkov emission angle θ is related to the beam tilt angle ϕ so that $\theta =90^{\circ }-\phi_{peak}$. The beam tilt angle of maximum Cherenkov emission was measured to be approximately 42.5°, corresponding to a Cherenkov emission angle of 47.5°. The expected Cherenkov emission angle according to Equation (1) is 47° for 12 MeV electrons in silica with n≈1.47 (using the refractive index at around 314 nm), which corresponds well to the measured peak emission.

In Figure 7, the emission peak luminescence is also shown at 314 nm, corrected for the loss of electrons due to beam tilting, as well as the increased path-length of the electrons in the material. The 314 nm RIL is thus expected to be fairly constant over all tested angles with those corrections in place. This is true within 20% for the measurements with the Ocean Optics spectrometer, and 35% with the monochromator. The decrease in observed luminescence at 314 nm for larger beam tilting angles remains not fully explained. There are some geometric effects that can play a role in the transmitted signal, such as the sample not being perfectly cylindrical or the doping profile not being perfectly homogeneous throughout the sample rod.

**Figure 8:**
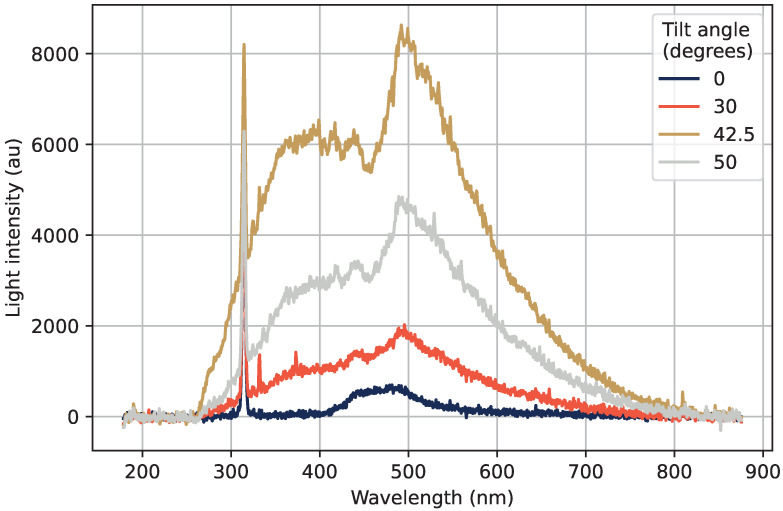
Emission spectra at different tilt angles, with the sharp emission peak of Gd^3+^ at 314 nm, as well as a wide Cherenkov emission spectrum with a maximum intensity at 42.5°.

The emission spectra acquired by the Ocean Optics spectrometer at different tilt angles are shown in Figure 8. The large increase in light emission due to Cherenkov radiation when the beam is tilted can be clearly seen, with a maximum in emission at approximately 42.5°. The exact shape of the measured optical spectra depends on many factors, where the response of the photon sensor is an important part. The radiation-induced attenuation (RIA) is also something that has an effect, since RIA has a spectral dependence and will modulate the measured emission spectra.

The emission structure at 0° in the region from 400 nm to 500 nm, which could also be discerned in Figure 3, has not previously been reported in the previous studies of this type of sample [1,3]. The tested sample in this study is the same one as was tested in [3], where very little emission can be seen in the region from 400 nm to 500 nm in the emission spectrum. This emission structure is further discussed in Section 3.5.

### 3.4. RIL at 314 nm

**Figure 9:**
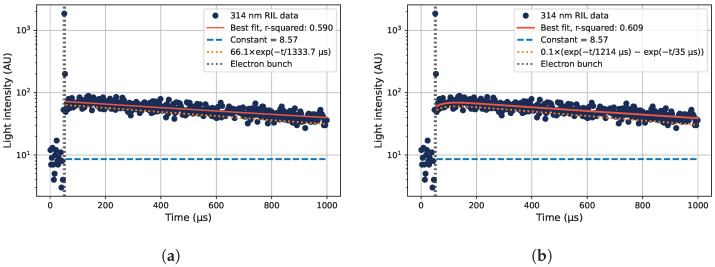
Measured decay curves of the 314 nm RIL of the Gd^3+^-doped sample, using 12 MeV electrons at a depth of 2 cm in acrylic. The time of the electron pulse is also shown. (**a**) Data fitted to one exponential decay component (orange dotted line) plus a constant representing the background level (blue dashed line), according to Equation (2). (**b**) The data fitted with one exponential decay component with an additional term to account for the initial increase in light emission intensity over time shortly after the electron bunch, described in Equation (3).

The main luminescence peak at 314 nm in Gd^3+^-doped silica glasses has a decay time measured around 1.35 ms in [1] and 1.80 ms in [5]. Fitting the measured luminescence signal after the electron pulse at 314 nm to an exponential decay function results in the fitted data shown in Figure 9a. The fit function was

(2) $$I\left(t\right)=Ae^{-t/\tau }+B,$$

where I(t) is the emitted light intensity as a function of time *t*, *A* is a constant scaling the amplitude of the light emission intensity, τ is the decay time of the luminescence, and *B* is a constant corresponding to the background signal level. The fitted value of the decay time is 1.33 ms, which is very similar to the decay time found in [1].

The fit is, however, not entirely satisfactory at times close after the electron pulse, where an increase in light emission is seen before the exponential decrease in light intensity is observed. A fit with a term accounting for the luminescence buildup is used instead to account for this behavior according to

(3) $$I\left(t\right)=C\left(e^{-t/\tau_{1}}-e^{-t/\tau_{2}}\right)+B,$$

where *I*, *t*, and *B* are described in connection with Equation (2), *C* is a constant governing the amplitude of the signal, $\tau_{1}$ is the decay time of the luminescence, and $\tau_{2}$ is the characteristic time constant describing the initial luminescence buildup. The data fitted to Equation (3) are shown in Figure 9b.

An interpretation of Equation (3) is that the luminescence of the decay described by the time $\tau_{1}$ is suppressed by a transient mechanism with diminishing strength described by a time constant $\tau_{2}$. This mechanism can be a transient radiation-induced attenuation (RIA), which strongly attenuates the induced light directly after the radiation pulse, then less after longer times. The transient absorption band at 4.2 eV caused by STEXs covers the 314 nm emission line [6], and can well explain the luminescence attenuation at times shortly after the electron bunch has hit the sample.

From the data in Figure 9, one can approximate the transient RIA behavior. As a first-order approximation of the RIA strength after the electron bunch, we assume that the luminescence described by $\tau_{1}$ is correct (although it might also be affected by a longer lasting time-dependent RIA). One can then extrapolate the fitted line in Figure 9b dominated by $\tau_{1}$ to the end of the electron bunch. The unattenuated (extrapolated) signal is then found to be ≈1.23× the attenuated (measured) signal there. Assuming a fiber length of 8 cm in the beam where RIA is induced, this corresponds to an initial RIA of approximately 11 dB/m after the end of the electron pulse for the case in Figure 9b.

**Figure 10:**
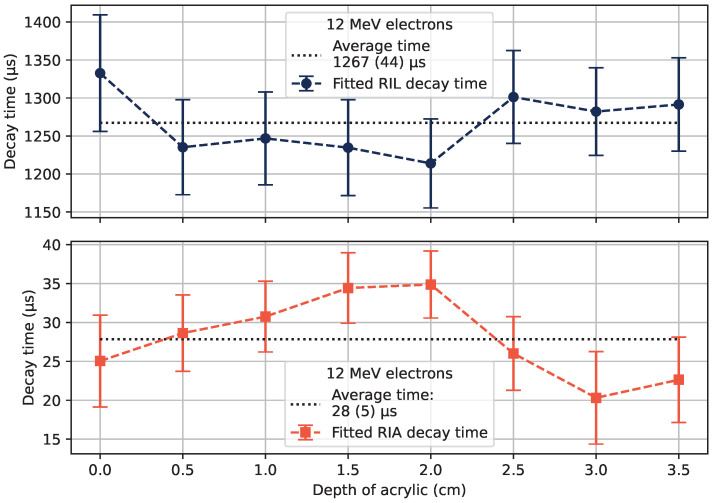
Fitted luminescence decay times (**top**), and transient RIA decay (luminescence buildup) times (**bottom**) at different depths of acrylic for 12 MeV electrons, with the estimated standard deviation of the fitted variable value as the error bars. The average of the measured values is shown with dotted lines, with the estimated standard deviations of the averages in parentheses.

Fits according to Equation (3) were made for 6, 12, and 20 MeV electrons at the different tested depths of acrylic. For 12 MeV electrons, the characteristic decay times are shown in Figure 10, with error bars signifying the estimated standard deviation of the fitted parameters based on the covariation matrix formed during the fitting procedure. The average values on the tested depths as indicated in Figure 10 are shown for all tested electron energies in Table 1. The calculated average values of the initial RIA are also shown in the table.

A further note is that the luminescence increase after the electron bunch is more pronounced at the depth of the maximum dose in acrylic than without any material over the sample, as well as more pronounced than points deeper in the acrylic. The reason for this could be that the transient RIA is dose-rate-dependent, so that the higher dose rate on the sample at the depth of the maximum dose in the material corresponds to a larger initial RIA. The trend of measured RIA strengths as a function of depth in acrylic does not, however, strictly follow the shape of the dose–depth curves. This could be masked by uncertainties in the individual fits, which could distort the shape of RIA curves as a function of depth. The measured values of $\tau_{2}$ and the RIA strengths rather vary around a specific level, which motivates the use of the average value in Table 1 to represent the generally observed trends.

### 3.5. RIL between 400 and 600 nm

**Figure 11:**
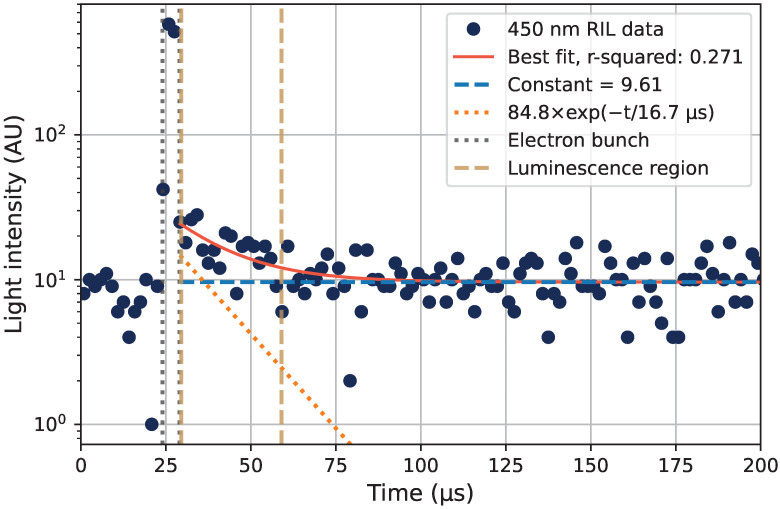
Luminescence decay measured at 450 nm, relative to the electron bunch time. The data were fitted to one exponential decay (orange-dotted line) plus a constant representing the background level (blue-dashed line), as given by Equation (2).

The optical emission structure seen at 0° in Figure 8 is further investigated in this section. The photon emission as a function of time, as measured using the monochromator setup at 450 nm, is shown in Figure 11, with the decay time τ= 16.7 μs when fitted to Equation (2) (one decay component). Other fitted decay times in the same wavelength region are shown in Table 2, where the best fits to the luminescence curves gave values from 10 μs to 15 μs.

**Figure 12:**
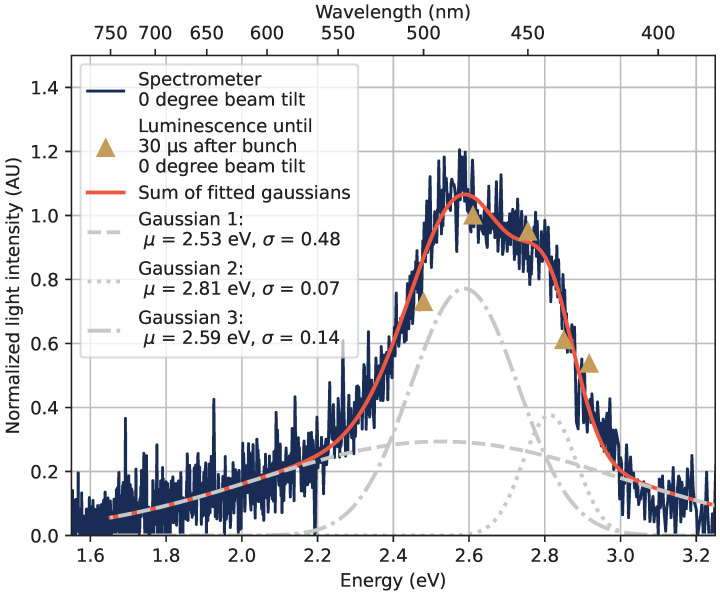
The observed peak in the optical emission spectrum between 400 nm and 600 nm. The structure is reproduced with an optical spectrometer as the blue line, and by summing the detected photons in the luminescence time region marked in Figure 11 by gold-dashed lines, resulting in the points marked by gold triangles in this figure. The spectrometer measurement was also fitted to a sum of three Gaussian components to recreate the peak shape.

The sum of detected photons in the region marked as *Luminescence region* in Figure 11 is compared, for the tested wavelengths, with the spectrum measured with the spectrometer. This is shown in Figure 12, where the emission spectrum from the spectrometer is the 0° spectrum from Figure 8. The 0° spectrum and the sum of detected photons in the *Luminescence region* coincide well with each other from 400 nm to 500 nm, suggesting that emissions in the luminescence region seen in Figure 11 are causing the peak observed using the spectrometer in Figure 12.

The measured peak is rather asymmetric, and likely does not consist of one static emission band. The spectrometer data were fitted to a sum of three Gaussian components in Figure 12, with a wide distribution centered at 2.53 eV (490 nm), and narrower peaks at 2.59 eV (479 nm), and 2.81 eV (441 nm). The position of the emission structure in the energy spectrum coincide well with previous measurements of the emissions from STEX centers, at, e.g., 2.4 eV in [6] and 2.8 eV in [9].

The blue-shift of the luminescent decay of the STEX were in [6] at 80 K found to be such that the luminescence peak center after 10 μs was observed at approximately 2.05 eV (605 nm), then shifting towards 2.4 eV (517 nm) over the course of tens of microseconds. This blue-shift in the STEX decay can cause some of the broadening and structure of the observed emission in Figure 12. Also note that the experiments in this study were performed at the ambient room temperature and not in a cooled down configuration.

In Table 2, a slight shift to longer decay times for shorter wavelengths could be estimated, but the fits made to the collected data in this experiment do not provide enough confidence to support such a blue shift. Further studies would be needed to confirm whether the emission peak observed in Figure 12 is composed of an emission center experiencing a blue-shift over time in the tested sample.

## 4. Conclusions

Sol–gel silica glass doped with Gd^3+^-ions shows great promise for applications in dosimetry as a point dosimeter using the RIL from ionizing radiation, which has an intensity proportional to the rate of deposited dose. In this study, the properties of the RIE under electron irradiation were studied under different irradiation conditions, allowing for the novel measurements of dose–depth curves, RIL decay times, transient RIA properties, and RIE spectra in this type of sample.

The RIL at 314 nm was found to be proportional to the deposited dose as a function of depth in acrylic for electron beams at 6, 12, and 20 MeV. The luminescence at 314 nm followed the dose–depth profile measured with a reference parallel plate ionization chamber dosimeter, while the Cherenkov radiation induced in the sample follows a different depth profile. To accurately represent the dose at varying depths in materials, it is thus necessary to only select the 314 nm RIL component.

The light emission was also investigated at a varying incidence angle of the beam, confirming a large presence of Cherenkov radiation in the measured RIE. A clear dominance of the Cherenkov light over the 314 nm RIL component was found for the angles allowing maximum Cherenkov light injection through the transport fiber.

The experiments were performed with two different setups: a commercial spectrometer and a monochromator with a PMT for light detection, which provided complementary data to each other. The systems offered different methods of discriminating the Cherenkov background, and only selecting the desired 314 nm RIL component.

With the monochromator and PMT setup, the decay time of the luminescence at selected wavelengths could be investigated. An emission band between 400 nm and 600 nm, with decay times of approximately 10 μs to 15 μs was observed. These emissions can be ascribed to the luminescent decay of STEX centers caused by the radiation on the sample. These emissions were not clearly observed in earlier tests with the same sample, which suggests an increase in these emissions with an increasing dose history. This should be further studied by obtaining emission spectra from this type of sample at increasing dose levels.

At the main emission line from Gd^3+^-ions in silica glass at 314 nm, the RIL intensity was found to first increase after the end of the impinging electron pulses. This is likely due to a transient RIA caused by the pulsed electron beam, which has a large instantaneous dose rate of approximately 280 Gy/s, attenuating the RIL signal at times close to the electron bunch. The characteristic decay time of the transient RIA was found to be approximately 20 μs to 30 μs. The transient RIA is ascribed to the STEX absorption centers observed in previous studies at 4.2 eV.

The decay time of the 314 nm RIL was measured to be approximately 1.3 ms for 6, 12, and 20 MeV electrons at various depths of acrylic, with some fluctuations in the exact fitted value. This decay time value is comparable with previously reported values of 1.35 ms in [1] and 1.80 ms in [5].

Further research into the formation of STEX centers, and the link to the property of increasing luminescence from the STEX-center decay as a function of radiation history on the sample, is needed. A study of the properties of the emission bands which form the RIE structure from 400 nm to 600 nm is also of interest.

## Figures and Tables

**Table 1:** Decay times of the luminescence at the main Gd^3+^-ion RIL at 314 nm under irradiation with electrons at a normal incidence angle. Fitted decay time values, transient RIA time constant, and RIA strength after the electron bunch are listed. The presented values are the average of the fitted ones at different depths of acrylic, with the estimated standard deviation in parentheses.

Electron Energy (MeV)	RIL Decay Time $\tau_{1}$ (μs)	Transient RIA Decay Time $\tau_{2}$ (μs)	Initial RIA Strength (dB/m)
6	1257 (43)	22 (4)	4.3 (2.1)
12	1267 (44)	28 (5)	7.2 (2.7)
20	1257 (44)	22 (9)	5.3 (2.4)

**Table 2:** Decay times of luminescence at different wavelengths in the emission region 425 nm to 500 nm during irradiation with 20 MeV electrons at a normal incidence angle without acrylic cover.

Wavelength (nm)	Decay Time (μs)
425	12.6
450	16.7
475	11.5
500	8.3

## Data Availability

The dataset that was analyzed to produce this publication is found in https://doi.org/10.23729/e7988ecf-d719-472e-9ad7-c66d0f7c427a.
